# Emotional contagion in adult English education: a self-narrative study of teacher–student interactions in Yangshuo County

**DOI:** 10.3389/fpsyg.2025.1549660

**Published:** 2025-01-28

**Authors:** Hongji Jiang

**Affiliations:** Guangxi Science & Technology Normal University, Laibin, China

**Keywords:** adult English education, emotional contagion, teacher–student interactions, self-narrative, emotional dynamics

## Abstract

This study aims to explore the emotional contagion dynamics between adult English learners and their teacher, focusing on the interplay of positive and negative emotional expressions across different proficiency levels and the pivotal role of learners as emotional initiators in shaping teacher–student interactions. Using a self-narrative study method, qualitative data were collected through interviews, informal exchanges, and reflective teaching logs involving 10 adult English learners at a private language training institution in Yangshuo County, China. As both the teacher and researcher, I captured and analyzed emotional exchanges, identifying three negative emotions: students’ anxiety, which triggered self-doubt in the teacher; students’ confusion, which led to the teacher’s disappointment; and students’ discontent, which evoked anger in the teacher. In contrast, two positive emotions were observed among intermediate and advanced learners: students’ calm fostering the teacher’s tranquility, and students’ happiness bringing the teacher relief. The findings challenge traditional assumptions by identifying adult learners as primary initiators of emotional contagion, with teachers often responding to these emotional cues. This study highlights how cultural norms, such as face-saving in China, and institutional pressures reshape teacher–student emotional dynamics. It provides practical implications for emotional regulation, culturally sensitive pedagogy, and the integration of communicative and traditional teaching methods, offering a foundation for future research on emotions in adult language learning contexts.

## 1 Introduction

The landscape of English language education in China has undergone significant shifts in recent decades. The 1990s reform era sparked a proliferation of English training institutions across major cities (Haidar and Fang, 2019; Hu, 2005). However, the rise of online resources, accelerated by the COVID-19 pandemic, led to the closure or downsizing of many institutions. This restructuring was further compounded by China’s Double Reduction Policy, introduced in 2021, which aims to reduce excessive academic workloads and regulate off-campus tutoring for K-12 students, indirectly impacting the broader tutoring industry (Guo, 2022). Amid these challenges, the language school MD (a pseudonym) in Yangshuo County, Guangxi, demonstrated resilience through its specialized focus on adult English education. Unlike prominent institutions affected by the policy, MD’s niche market offered significant advantages. This distinction arises from its emphasis on adult education, fostering distinctive teacher–student interactions that adapt particularly well to policy shifts. Such a specialized focus warrants attention, as it provides valuable insights into the dynamics of adult English education in China, a field with limited existing research.

Globally, scholars have extensively examined teacher–student dynamics in foreign language education (Smit et al., 2022). Studies on emotions within language education context have explored various themes, including teacher dynamics, peer influences, and the intricate mechanisms underlying positive emotions like enjoyment and happiness (Dewaele and Li, 2021; Moskowitz and Dewaele, 2021; Shao and Parkinson, 2021; Talebzadeh et al., 2020). Despite the wealth of research on emotions in teacher–student relationships (de Ruiter et al., 2021), empirical investigations specifically addressing emotional contagion between teachers and adult students remain scarce, especially in private language schools in China. Moreover, a notable gap emerges as research often overlooks negative emotions, particularly in the context of adult learners and teacher emotional contagion. Understanding negative emotions such as anxiety and frustration in adult education could yield valuable insights for emotional intelligence and pedagogical practices.

## 2 Literature review

### 2.1 Adult language learners

Adult learners in this study differ significantly from college students due to their work experience and the practical goals driving their English learning. For many, effective English communication is essential for career advancement or community integration in China (Yu and Liu, 2022). Unlike younger learners, adults rely on their rich life experiences and mature cognitive abilities, which can support their language learning process in unique ways (Bigelow and Schwarz, 2010). However, for adults with a weak foundation in English, these strengths may be counterbalanced by heightened anxiety about making mistakes, which can undermine their confidence and willingness to take risks essential for language acquisition (Griffiths and Slavkov, 2021; Schuetze and Slowey, 2013).

Research on adult learners’ motivations reveals their engagement with language programs stems from an intricate interplay of personal and professional aspirations. For instance, Huang (2020) highlighted how enhancing self-efficacy—through success experiences, verbal persuasion, and managing emotional states—can significantly improve adults’ learning interest and proficiency. Similarly, Koiso (2003) study of Japanese adult English learners provided a comparative analysis of motivational differences across age and gender, shedding light on the diversity of goals and attitudes in adult learning contexts. Zhu (2019) underscored the role of language learning strategy training in fostering autonomy and sustainability among adult learners, while Ma (2018) evaluated how role-playing methods can enhance English communication skills in professional settings.

Although these studies contribute to understanding adult language learners’ motivations and strategies, there remains a noticeable gap in research specifically examining adult English learners in China’s unique sociocultural and educational context. In particular, the role of emotional contagion in shaping teacher–student dynamics and influencing adult learners’ engagement and their relationships with teachers has yet to be thoroughly investigated.

### 2.2 Emotion contagion

The role of emotion in foreign language education has gained prominence in recent years, with scholars increasingly recognizing its impact on motivation, engagement, and overall language acquisition (Bielak and Mystkowska-Wiertelak, 2020; Dewaele and Li, 2020; Gregersen et al., 2014; Li et al., 2018; Liu and Fan, 2021; Talebzadeh et al., 2020). Negative emotions like anxiety can hinder learning outcomes, while positive emotions such as pleasure are associated with enhanced performance (Horwitz, 2001; Li and Wei, 2022). Beyond individual emotions, studies have explored the interplay between emotions and motivational trajectories in language learning. For example, Shao et al. (2019). demonstrated how discrete emotions can influence cognitive functioning and task performance, while Pavelescu (2023) explored their relationship with motivation and willingness to communicate. Yu et al. (2022) further highlighted how emotion regulation strategies mediate the link between motivation and burnout.

The emotional experiences of teachers are equally critical, influencing classroom dynamics and teaching effectiveness (Benesch, 2017; Yin et al., 2019). Research into teacher emotions has focused on strategies for regulating anxiety and preventing burnout (Shen, 2022). Studies on novice teachers emphasize their emotional sensitivity and enthusiasm, which can both enrich and challenge the learning environment (Caspersen and Raaen, 2014; Schatz-Oppenheimer and Dvir, 2014). Furthermore, the interplay between emotion management and social bonds has been investigated in the context of early career science teachers, shedding light on the impact of emotion management on teacher–student relationships (Bellocchi, 2019). Understanding the emotional exchanges between teachers and students is vital for supporting teacher wellbeing and fostering positive interactions (Golombek and Doran, 2014).

However, the studies of learner emotions and language teacher emotions seem to be “separate,” and few studies pay attention to the interaction of teacher and student emotions (Fredrickson, 2003). Emotional contagion, defined as the automatic and unconscious transfer of emotions between individuals, has emerged as a key area of interest (Hatfield et al., 2014). This phenomenon plays a significant role in teacher–student interactions. For instance, Talebzadeh et al. (2020) examined how happy emotions were transmitted between Iranian teachers and students through verbal and non-verbal cues, demonstrating the synchronization of behaviors in classroom settings. Similarly, Dewaele and Li’s (2021) study of Chinese college learners highlighted how their emotional perceptions of teachers influenced their engagement and classroom experiences.

Despite these insights, several gaps remain in the literature. Existing research tends to focus on positive emotions, overlooking the importance of negative emotional exchanges in shaping the learning environment. Additionally, cultural influences on emotional contagion are underexplored, and learner perspectives are often marginalized in favor of teacher-centered analyses. Furthermore, the dominance of quantitative methods limits a deeper qualitative understanding of emotional experiences. Addressing these gaps through studies like the present one can offer a more nuanced view of emotional contagion in foreign language education.

### 2.3 Conceptual framework

This study integrates three key theoretical perspectives—Control-Value Theory (CVT), Role Theory, and the concept of face culture in China—to examine the bidirectional emotional contagion between teachers and adult learners in a private language education context. These perspectives collectively provide a multidimensional lens to analyze the emotional dynamics observed in the study.

#### 2.3.1 Control-value theory (CVT)

Control-Value Theory (Pekrun, 2006) posits that emotions in academic settings arise from the interaction of two factors: the individual’s perceived control over a task and the value they attribute to it. In this study, CVT will help to explain the emotional dynamics among adult learners. For beginner learners, limited control over learning activities, coupled with high task value (e.g., career advancement), often leads to negative emotions such as anxiety and confusion. Conversely, as learners gain proficiency, their increased sense of control fosters positive emotions like pride and happiness. This theory provides a robust framework for understanding the evolving emotional experiences in adult language learning.

#### 2.3.2 Role theory

Role Theory emphasizes the societal expectations and norms associated with specific roles, such as teacher and student (Biddle, 1986). In the Chinese educational context, these roles are influenced by hierarchical cultural norms, where teachers are traditionally viewed as authority figures. However, adult learners, particularly in private education settings, challenge this dynamic due to their financial investment and professional experience. This tension redefines traditional teacher–student roles, influencing emotional exchanges. For example, discontent is often expressed indirectly, such as through class transfers, reflecting the interplay of cultural expectations and individual agency. Moreover, teachers in this context face the dual challenge of balancing their traditional role as educators with the service-oriented expectations of adult learners, further intensifying emotional labor.

#### 2.3.3 Face culture in China

Face culture, deeply rooted in Chinese society, pertains to maintaining social harmony and preserving one’s reputation (Ho, 1976). This cultural dimension significantly shapes teacher–student interactions, particularly in managing emotional exchanges. For example, students may suppress direct criticism of teachers to avoid causing embarrassment, opting instead for subtle strategies like disengagement or withdrawal. Similarly, teachers navigate institutional pressures while striving to maintain their professional “face,” balancing emotional labor and pedagogical objectives. Face-saving behaviors often dictate indirect forms of communication, such as students changing classes rather than confronting their teachers directly, illustrating how cultural norms influence emotional expressions and decisions.

#### 2.3.4 Integration of theories

The integration of CVT, Role Theory, and face culture offers a multidimensional lens for understanding emotional contagion in teacher–student interactions. CVT highlights how learners’ perceptions of control and task value influence emotional responses, such as anxiety from a mismatch between perceived ability and task demands. Role Theory situates these emotions within evolving teacher–student dynamics, where adult learners’ life experiences and financial investment challenge traditional hierarchies. Face culture adds a cultural perspective, emphasizing harmony and indirect communication, as seen when students avoid direct criticism to protect their teacher’s “face.” Together, these theories illuminate how cultural, societal, and individual factors shape emotional dynamics in adult language education.

This study aims to bridge this gap by conducting a qualitative self-narrative inquiry, collecting data through interviews and informal exchanges with adult English learners. Insights from this study could inform teaching strategies and emotional support mechanisms tailored to the unique needs of adult language learners. The research questions guiding this study are:

1. What are the emotional contagion dynamics, including negative and positive exchanges, that emerge between a novice teacher and adult English learners in a private language school setting?
2. How do cultural norms (face culture), role expectations, and emotional regulation strategies (within the framework of control-value theory) influence the emotional contagion dynamics between a novice teacher and adult students in the context of Chinese education?

## 3 Methodology

### 3.1 Research context

China faces a significant demand for adult English language training, yet the availability of institutions often falls short of meeting this need. Yangshuo County, known for its picturesque karst landscapes and outdoor activities, provides an ideal backdrop for adult learners seeking immersive language experiences. MD, established in 2001 in Yangshuo, Guilin, is a specialized English language institution that integrates language education with the natural beauty of its environment. In addition to English courses, MD offers Chinese language classes for international students, promoting informal language exchanges between international and Chinese students. Conveniently situated in an urban area with accessible transportation, MD had around 60 students and 20 teachers, both Chinese and foreign, in 2021 and 2022, influenced by the pandemic. The institution maintains small class sizes, typically ranging from 5 to 10 students, and provides full-time study opportunities with dormitory and canteen facilities. The author, a 35-year-old university English instructor and doctoral candidate, joined MD as a part-time teacher. While the doctoral research focuses on secondary education and is unrelated to this study, the teaching role at MD offered an opportunity to explore adult English education in a distinctive and immersive context, despite lacking prior experience teaching working adults outside a university setting.

### 3.2 Research participants

This study is based on my teaching practice at MD, an adult English training school in China, where I worked part-time during my doctoral studies. MD is one of few full-time English training institutions in China, with students assigned to one of seven proficiency levels. Chinese teachers primarily handle levels 0 to 3, while native English-speaking teachers predominantly teach levels 4 to 6. The 10 participants were selected based on convenience sampling, as they were enrolled in my Level 1 class. After a month of interacting with these students, I noticed a strong emotional dynamic, where I, as a novice teacher, was “infected” by their emotions. This led me to use purposeful sampling to focus on those students who best illustrated the emotional contagion process in the classroom context.

This study focuses on 10 students from my Level 1 class, which I began teaching in May 2021. The class consists of 10 adult learners (see Table 1) with similar proficiency levels, advancing one level every 2 months on average. While I also taught a Level 0 class with three students, the primary focus of this study is on the Level 1 students. The students at MD are working adults, older than typical college students, from diverse professional backgrounds such as foreign trade, programming, and nursing. Studying at this institution for just one or 2 months is often seen as both ineffective and costly. Consequently, most students opt for longer programs of over 10 months, which offer more favorable tuition rates and VIP benefits. However, this commitment comes with significant sacrifices: working individuals often have to quit their jobs, and families are frequently separated from their loved ones for extended periods. In this study, adult English learners described the lengthy deliberations they underwent before committing to study English at this training school, ultimately making the decision to fully dedicate themselves to their language learning journey. One student shared that he had been contacted by the school’s sales representative 2 years prior to enrolling. After much consideration, he decided to leave his position as a car sales representative to pursue English studies.

**Table 1:** Background of research participants.

Number	Sex	Age	Occupation	Comes from
S1	Female	32	Full-time mother	Guangdong
S2	Female	35	Full-time mother	Guangdong
S3	Male	47	Freelancer	Guangdong
S4	Female	26	French translator	Guangdong
S5	Female	37	Full-time mother	Guangdong
S6	Male	30	Unemployed	Shanghai
S7	Male	33	Skiing instructor	Jilin
S8	Male	36	Unemployed	Hunan
S9	Female	35	Unemployed	Beijing
Wei Ying	Male	33	Photographer	Beijing

“Unemployed” participants were either in career transitions or temporarily not working during the study.

Wei Ying (a pseudonym), a 33-year-old photographer, was chosen as the main participant due to our frequent and meaningful interactions both inside and outside of class. Sharing similar ages and interests, our regular communication provided valuable insights into the emotional experiences and challenges faced by adult learners. Although my Level 1 class was diverse in terms of age, profession, and educational background, Wei Ying was selected as the primary subject because of his unique interactions with me and his role in illustrating the emotional contagion process. Wei Ying’s active participation in class also made him a key example of the emotional dynamics between teacher and student in this context. This purposeful selection aligns with the qualitative approach, which emphasizes in-depth, meaningful interactions rather than generalizable trends.

### 3.3 Research method

This study uses a qualitative case study design and adopts a self-narrative research framework to explore the evolving emotional interactions between teachers and students in adult English education. The self-narrative approach is particularly suitable for studying emotion contagion, as it enables the researcher to reflect on personal experiences, emotional responses, and their influence within the teacher–student dynamic (Hayler, 2012). This method allows for an in-depth examination of emotional cues, the triggers of emotions, and their perceived effects on the language learning process. By using self-narrative, the researcher can gain a rich, subjective understanding of how emotions flow between teacher and students, which is crucial in exploring emotional contagion in the classroom context (Kennedy-Lewis, 2012).

The choice of self-narrative is justified for this study due to its unique ability to capture the subjective nature of emotional experiences, particularly in adult language learning settings where personal interaction and emotional exchange are central to the learning process. It offers an insider’s perspective, thus providing valuable insights into the emotional dynamics of the teaching-learning relationship. In addition, the approach facilitates a nuanced understanding of the emotional changes that occur over time, as the researcher can analyze how emotional experiences are triggered, modified, and shared between teacher and students.

To ensure rigor, this study employs triangulation to strengthen the validity of the findings. Data collection spanned from May 2021 to March 2022, using multiple sources of evidence: (1) informal communication with adult students, (2) semi-structured interviews with students, and (3) teaching logs and reflections. The semi-structured interview aimed to explore participants’ experiences studying English at the training institution. The primary question posed was: “Can you talk about your experiences studying English here?” This open-ended question encouraged participants to share their thoughts, emotions, and reflections freely, providing rich qualitative data. Teaching reflection logs were written intermittently, focusing on moments when the teacher/researcher experienced strong emotional responses or gained significant insights about classroom interactions. These reflections were not recorded daily but were prompted by impactful teaching experiences, notable emotional exchanges with students, or moments of personal growth. These varied data sources provide a comprehensive view of the emotional exchanges in the classroom, supporting the validity of the study’s conclusions (Cohen et al., 2017). Data was analyzed through thematic analysis, with the NVivo qualitative software aiding in the categorization and analysis of emotional expressions. By triangulating these diverse data sources, the study enhances the depth and reliability of its findings.

The use of self-narrative as a method is in line with ethical research practices, as it allows for a reflective, transparent, and responsible examination of personal emotional experiences, while minimizing the risk of harm or bias (Ellis et al., 2011). Ethical approval for this study was obtained from the relevant institutional review board, ensuring that all participant interactions were conducted in accordance with ethical guidelines. Informed consent was obtained from all participants, and confidentiality was maintained throughout the study. Participants were fully informed of their right to withdraw from the study at any time without penalty, ensuring voluntary participation. Feedback on the findings was provided to all participants after completion of the study.

### 3.4 Data analysis

This study employed a combined approach of thematic and narrative analysis to explore the emotional experiences in adult English education. Thematic analysis identified recurring themes in the narratives, revealing patterns of emotional expression, while narrative analysis examined how individuals construct meaning through their stories.

To ensure reliability, I analyzed qualitative data collected through interviews, informal communications, and teaching logs using NVivo software. A coding scheme was developed based on the research questions and key emotional constructs, such as anxiety, confusion, and emotional regulation. One participant’s data was initially coded to test the framework and ensure consistency. Emotional expressions were coded under corresponding categories (e.g., “anxiety,” “confusion,” “calm”) and reviewed iteratively to refine the scheme and ensure alignment with the theoretical frameworks.

The coded data were organized into broader themes using NVivo’s thematic analysis tools. Recurrent patterns and comments, such as the tension between teaching methods and learner preferences, were categorized under themes like “discontent” or “happiness.” Using a naturalistic, inductive approach, themes were arranged from general emotional dynamics to specific subcategories (e.g., anxiety at the beginner level vs. calm at intermediate level). These themes are presented systematically in the findings section, with each theme supported by illustrative quotes and examples to demonstrate how they emerged from the data.

Finally, narrative analysis mapped emotional trajectories in teacher–student interactions, highlighting key emotional turning points and their impact on teaching strategies. The findings are organized to emphasize the interplay between teacher and student emotions, showing how specific emotional episodes contributed to broader patterns. This dual approach ensured both methodological rigor and depth in understanding emotional contagion in adult English education, while also enabling a clear presentation of the data to capture the complexity of the emotional dynamics observed.

## 4 Findings

Thematic analysis identified five distinct emotional dynamics in teacher–student interactions: anxiety and self-doubt, confusion and disappointment, discontent and anger, calm and tranquility, and happiness and relief. Students’ emotions come first, followed by teachers’ emotions, a process that aligns with the definition of emotional contagion—"the tendency to automatically mimic and synchronize expressions, vocalizations, postures, and movements with those of another person, and consequently, to converge emotionally” (Hatfield et al., 1993).

**Figure 1:**
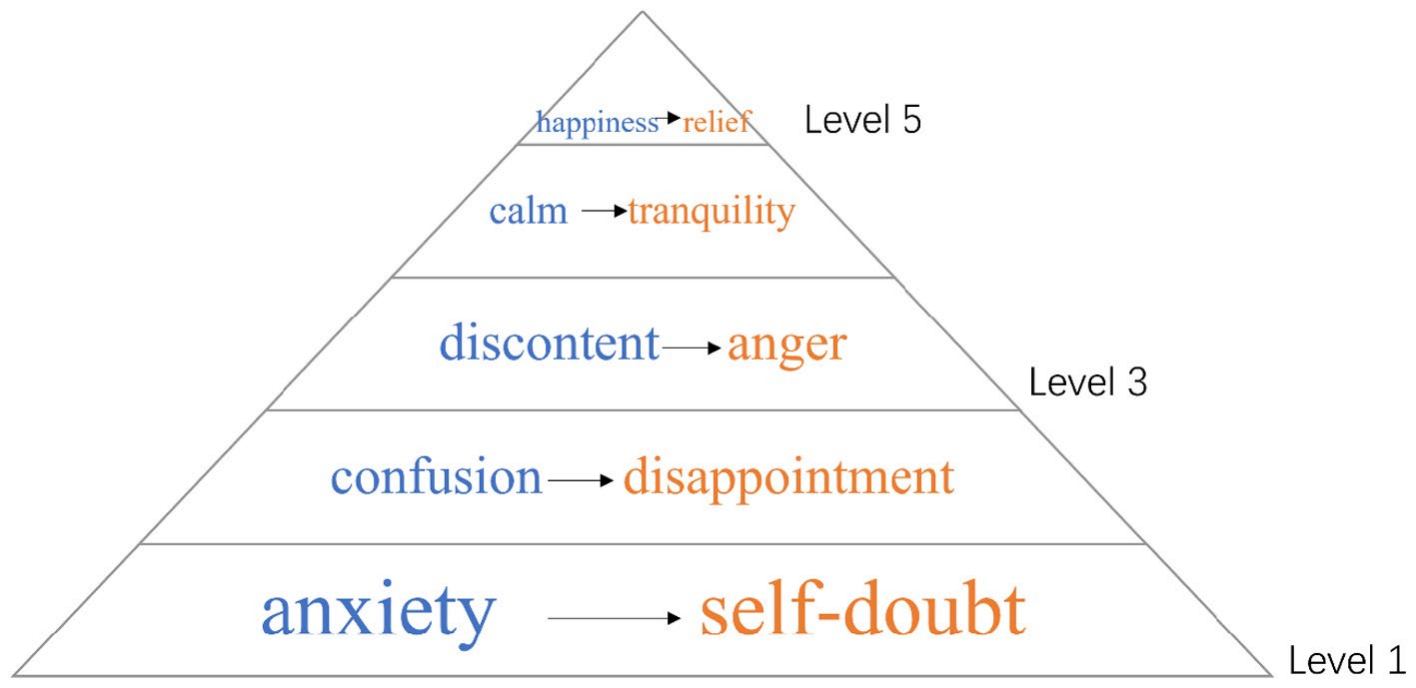
Emotional contagion dynamics in adult teacher–student interactions.

Both teachers and students are emotional initiators and receivers, influencing and “infecting” each other in traditional Chinese secondary schools. However, in the adult education context of this study, the dynamic shifts: teachers, aiming to maintain good relationships with students, often become the recipients of emotions. The diagram below (Figure 1) illustrates the five emotional dynamics observed in teacher–student interactions, highlighting how emotions initiated by students influence corresponding emotional responses in teachers. Blue represents students’ emotions, while orange represents the teacher’s emotions.

Section 4.1 focuses initially on the negative emotional dynamics—anxiety and self-doubt, confusion and disappointment, and discontent and anger—primarily observed among beginner learners, examining their impact on teacher–student interactions. Section 4.2 shifts to explore the roles of teachers and students in these exchanges and discusses the two positive emotional dynamics—calm and tranquility, and happiness and relief—which emerge as learners progress to intermediate or advanced levels.

### 4.1 Muted resistance: tracing the emotional origins of students’ requests for class transfer

This section examines the emotional exchanges between a novice teacher and adult learners through three key dynamics: anxiety and self-doubt, confusion and disappointment, and discontent and anger.

#### 4.1.1 Students’ anxiety and the teacher’s self-doubt

All my adult students in the level one class expressed discomfort, nervousness, and anxiety during their English studies. Balancing family responsibilities and demanding work schedules left them with little time to dedicate to learning, often leading to feelings of inadequacy and frustration. This anxiety was particularly pronounced among learners with weak foundational skills in English, who struggled with even basic tasks and expressed doubts about their ability to improve. These emotional struggles directly “infected” the novice teacher (myself), as I often felt overwhelmed by their visible discomfort and doubted my ability to meet their expectations or provide effective support. One adult learner described her frustration and anxiety:

*“I am a mother of two children. Now my son is five, and my daughter is four. Before I came to MD, I was a stay-at-home mother. So far, I am not satisfied with my English level.”* (S2).

This anxiety was amplified by their high self-expectations and the perception of slow progress. Another learner shared her experience of repeated efforts yielding minimal improvement:

*“I asked my classmates about the meaning of a certain word during class, and I found that the teacher had just talked about this word yesterday. It felt like a sharp weapon for killing people; it would make me uncomfortable.”* (S3).

Wei Ying’s anxiety about memorizing vocabulary exemplified the tension many learners felt. He lamented:

*“Teacher, I always have trouble remembering words and forget them after memorizing them. What should I do? Is there any way to memorize words quickly? Schools should devise a way to memorize words quickly so they can compete in the marketplace.”*

In our conversations, I suggested mnemonic techniques, such as associating English words with Chinese homophonics. For instance, I proposed using “pregnant” as “pu le ge gen tou” (Pinyin, literally meaning “took a tumble”) and “ambulance” as “an bu. neng si” (Pinyin, literally meaning “I cannot die”). Wei Ying initially agreed and experimented with these methods. Later, we discussed software options for memorizing words, and he suggested that schools adopt multimedia devices for more effective learning. He commented:

*“In this day and age, schools are so backward that they should use multimedia devices. In this way, we can use software to memorize words much more effectively.”*

However, I later realized that Wei Ying’s feedback contained an implicit critique of my teaching methods. During one conversation, he excitedly shared his experience of attending a foreign teacher’s elective course:

*“The foreign teacher used pictures to teach us food words. I still remember.”*

This indirect comparison highlighted his dissatisfaction and anxiety with my vocabulary instruction. In response, I explained my reasoning:

*“Foreign teachers teach you once, but I have to catch up with the progress. Otherwise, how can I finish the textbook?”*

Despite this explanation, Wei Ying’s anxiety persisted. During a casual conversation with classmates, he remarked:

*“When I was a child, my English teacher used Chinese to teach English, such as ‘pregnant’ or ‘education’ (‘ai jiu kai xin’ in Pinyin, literally meaning ‘love is happy’). This Chinese memory method has hurt me for a lifetime.”*

Hearing this, I felt a mix of frustration and self-doubt. Reflecting on these exchanges, I began questioning whether my approach—focused on structured lesson plans—aligned with student expectations.

*“No method works. You still have to memorize it by rote,”* I told him eventually.

Anxiety among learners stemmed from unmet expectations, perceived inefficiencies in learning methods, and frustrations with progress. This incident with Wei Ying led me to reconsider my teaching strategies, prompting a balance between creative and traditional approaches, such as incorporating more direct vocabulary instruction.

#### 4.1.2 Students’ confusion and the teacher’s disappointment

The emotional dynamic between students and the teacher often revealed a pattern where students’ emotional states directly influenced the novice teacher’s feelings. Specifically, learners’ confusion—rooted in uncertainties about their goals or learning methods—frequently triggered feelings of disappointment in the teacher. This emotional state was particularly prevalent among those navigating significant life transitions. For example:

*“I face many new starts, new challenges, and do not know where to go.”* (S4).

Housewives and professionals alike articulated the tension between their aspirations and current realities:

*“As a mother, I have also experienced the confusion of all stay-at-home mothers. How to take good care of your children and at the same time keep learning without being eliminated by society? How can we continue to realize our former ideals and wishes?”* (S2).

*“I have been a housewife for four years. After my children went to school, I became confused and did not know what I should do or what I could do. I think financial independence is important for any woman.”* (S5).

In the classroom, confusion often surfaced in requests for clearer instruction. Wei Ying’s plea underscored the frustration many students felt:

*“Teacher, please teach us grammar, because we do not know whether what we say is wrong or right.”*

Initially hesitant, I decided to teach grammar explicitly. I drew a table of 12 tenses on the board, explaining how to remember them systematically. After class, Wei Ying expressed his relief:

*“It turns out that tense is time and state. After hearing what you said, I suddenly became enlightened.”*

However, confusion resurfaced as Wei Ying struggled with the nuances of grammar rules, such as the distinction between past simple and present perfect tenses:

*“When to use the past simple tense and the present perfect tense? There is no clear line.”*

This ongoing confusion mirrored a broader challenge among students, who often skipped morning and evening self-study sessions. Reflecting on this, I noted in my journal:

*“Sad, (they) do not arrive on time for morning and evening self-study, do not come, do not study hard, do not read or memorize.”*

Learning English as a body of knowledge seems to be ingrained in the habits of Chinese learners. They often expect to acquire concrete knowledge in class, whether it involves new vocabulary or grammar rules. In my class, I incorporated many activities, but this sometimes left students feeling they had “gained nothing” after class. While they found the class interesting and fun, they felt that they had not truly learned anything substantial. This reaction could be attributed to their confusion about making rapid progress or to their deeply rooted learning habits developed over many years in the Chinese education system. One of my students expressed confusion after attending a native English-speaking teacher’s class, saying, *“The foreign teacher just let us go to the street and talk to foreigners in English.”*

The learners’ confusion resonates with existing research on emotional contagion in adult learning, highlighting the tension between extrinsic motivations, such as career advancement, and intrinsic uncertainties about learning goals or methods. This emotional ambivalence was particularly evident among learners undergoing significant life transitions, where their need for clear guidance often clashed with feelings of disorientation regarding their progress or approach to learning. Many were uncertain about whether to adopt traditional or activity-based learning methods, reflecting a broader struggle between familiar habits and new approaches. Additionally, confusion arose over their preference for Chinese native-speaking teachers or English native-speaking teachers. Chinese teachers, often likened to coaches, guided students through repeated text reading, requiring little active English speaking in class. By contrast, English native-speaking teachers encouraged more spontaneous interaction, which some learners found disorienting or unproductive. I, as a novice teacher, teach them like an English native-speaking teacher due to my teaching philosophy.

#### 4.1.3 Students’ discontent and the teacher’s anger

Students’ discontent in this study arose from a mismatch between their expectations and the teacher’s chosen teaching methods or pace of instruction. This discontent, often conveyed through non-verbal cues—such as the class transfer incident highlighted in this study—triggered feelings of anger in the novice teacher. The class transfer event involved three students, who requested to leave my class and be reassigned elsewhere. This initially left me confused and then angry. I had no knowledge of the transfer until my director asked me to meet him in his office. When I inquired about their reasons for transferring, S6 offered a self-deprecating explanation:

*“We might just be that local dish.”* (S6).

The term “local dish” metaphorically reflected S6’s feelings of inadequacy and struggle to keep up with the pace of learning. This feedback was both humbling and revealing. S6 also clarified:

*“I do not want to change your teaching methods because you are professional. First, I do not really want to learn phonetics. That’s the first thing. Second, I do not think your teaching style is bad; it’s just that I struggle to keep up. We all know you are very skilled, absolutely top-notch, but it’s just that we are not as proficient.”*

While this reassured me about my professionalism, it also highlighted the gap between my instructional approach and the students’ readiness or preferences.

Another incident involved frustrations with the “activity-centered” teaching method I implemented. My aim was to focus on communicative skills by designing activities such as role-playing, rapid-fire questions, and improvisations. However, students at an elementary level often found these tasks too demanding. This mismatch led to my growing anger:

*“In fact, this (designing and organizing these activities) makes very tired, and because they do not study after class, do not memorize the assigned things, and do not complete the activities in class.”*

In response to this feedback, I sought guidance from the head of the teaching department. The director suggested that I observe other Chinese teachers’ classes and try to adjust my own. After struggling with the situation, I finally decided to change my teaching approach and wrote a message to Wei Ying to ask for his opinions:

*“The activity-centered teaching concept seems not suitable for everyone. First, everyone’s consciousness is not high. The assigned tasks are not done, and what should be memorized is not memorized. As a result, the activities are not thorough and there may be no achievements. Second, due to low self-awareness, the level cannot be improved, the activities are not thorough, and there is no sense of gain. The third is that adults are still used to ‘harvesting’ certain knowledge, such as vocabulary and grammar. The new teaching method will be knowledge-centered and return to the traditional PPP model. First, teach new words, new grammar, and new knowledge points, practice vocabulary by making sentences or dialogues, and do homework.”*

Wei Ying’s response further emphasized the need for more organized and beginner-friendly instruction:

*“Everyone hopes to capture more knowledge in a shorter period. Perhaps it is because we are too lazy to memorize words, rather than an instructional design problem. Because the foundation is too poor, we cannot capture more knowledge in games or activities. Information, unable to participate. The activity is very particular about participation. If I cannot participate, there is nothing I can do. Maybe our foundation is too poor.”*

This dialog with Wei Ying reinforced my decision to adapt my teaching strategies. I realized that while activity-based methods work well for advanced learners, elementary-level students often benefit more from structured, incremental learning approaches. Consequently, I shifted to a PPP (Presentation, Practice, Production) model to better align with student needs.

The emotional toll of these adjustments was significant. At times, I felt frustrated and unappreciated, particularly when students did not follow through with self-study or homework assignments. However, these experiences also taught me valuable lessons about flexibility and the importance of meeting students where they are in their learning journey.

Reflecting on these events, I documented in my teaching journal:

*“The activity-centered approach, while theoretically sound, does not align well with the needs of students who lack foundational skills or intrinsic motivation. Moving forward, I will prioritize a hybrid approach that combines structured lessons with manageable activities to ensure progress without overwhelming learners.”*

All 10 students I taught exhibited similar emotions to varying degrees simultaneously. Among them, the emotions of “confusion and disappointment” were particularly prominent, with Wei standing out as the most notable. In “discontent and anger,” the emotions of three students, led by S6, were especially striking. These shared emotional expressions were verified through multiple sources. For instance, after the class transfer incident, I approached Wei for confirmation. This is one of the reasons why this study uses Wei as the central focus. As their English proficiency improved—for example, advancing from Level 1 to Level 3—their negative emotions gradually diminished, while their positive emotions steadily increased.

### 4.2 Getting better: the growth trajectory of the teacher and students

Over the course of the study, the dynamics between the teacher and students shifted from frustration and self-doubt to mutual growth and emotional resilience. This transformation highlights how emotional exchanges in the classroom can evolve alongside improved academic performance and personal confidence.

#### 4.2.1 Students’ calm and the teacher’s tranquility

For many adult learners, mastering English is a lengthy and demanding process, yet their persistence often yields noticeable progress. As my students gradually cultivated a sense of calm and determination in their learning journey, this emotional state often transferred to me as their teacher, fostering a shared sense of tranquility in our interactions. As one student, S8, reflected during an informal conversation, *“You cannot rush to learn English.”* This sentiment resonated with many others in the class, who began consoling each other with similar words of encouragement as their confidence grew. After 4 months, most students had advanced to Level 3, a milestone that brought a sense of accomplishment. As S10 shared*, “Now, I have become more and more familiar with foreign students, and I dare to chat casually. Although the topics are not rich because of my limited vocabulary, I know that one day I will be able to chat as much as I want.”*

The growth of my students was accompanied by my own development as a teacher. Initially, I struggled with self-doubt and frustration, often questioning whether my communicative, activity-centered teaching methods were appropriate for adult learners with weak foundations. Through reflection and adaptation, I gradually shifted toward a knowledge-centered approach. In my teaching diary, I wrote, *“I have resolved the contradiction between the communicative teaching philosophy I uphold and the basic and actual needs of students, making a compromise between ideals and reality.”* This change involved integrating more focused instruction on key vocabulary and grammar, supported by authentic materials and structured tasks like weekly dictations and grammar explanations.

Wei Ying, one of the students, embodied this sense of calm and determination. Initially overwhelmed by the difficulties of memorizing vocabulary, he chose to persevere through rote memorization. *“Sometimes I would spend five hours memorizing 70 words. After 2–3 months, my word memorization notebook was almost 5 cm thick. I was able to communicate with foreigners using individual or multiple words, which gave me a great sense of accomplishment,”* he shared during an interview. This persistence eventually gave him the confidence to participate in a school speech competition. As he recalled*, “Just before my speech, when I was extremely nervous, the encouraging look teacher Melissa gave me immediately calmed me down. At the moment I won the prize, teacher Maria told me: ‘Great job, I’m so proud of you!’ The joy and excitement in my heart are indescribable.”*

#### 4.2.2 Students’ happiness and the teacher’s relief

In the latter stages of the study, the classroom atmosphere was characterized by a shared sense of happiness and relief. As students began to experience the rewards of their hard work and progress, their joy became contagious, alleviating the teacher’s earlier feelings of pressure and fostering a mutual sense of accomplishment and ease. S2 noted, *“Every Thursday there will be free discussions, singing, guessing words, and all kinds of social activities. By participating in these activities, I can not only meet many interesting people but also improve my social skills.”* Similarly, S7 reflected, *“Through memorizing words every day and communicating with foreign teachers, I can now freely communicate with foreigners and even joke with them, truly integrating English into my life.”*

For some students, the recognition of their efforts provided additional motivation. S9 vividly described their reaction upon being named an “Excellent Summer Class Teaching Assistant,” which came with 3 weeks of free classes: *“When I learned about this news, I felt like I had won the lottery and was happy for a long time.”* Such moments reinforced the connection between external validation and internal growth, driving students to continue striving toward their goals.

As a teacher, I found immense joy and relief in witnessing my students’ achievements. One particularly memorable moment came when Wei Ying advanced to Level 5. He messaged me to share the news, and I wrote in my diary, *“Adult English teachers must think in others’ shoes and put themselves in the position of beginners from time to time. Beginners need encouragement, motivation, and a sense of accomplishment.”*

This mutual growth between teacher and students underscores the transformative potential of adult English education. As students gained confidence and skills, I also evolved as an educator, learning to balance the demands of teaching with the emotional needs of my learners.

## 5 Discussion and implications

### 5.1 Adult English learners as initiators of emotional contagion: insights from control-value theory

This study explores the bidirectional emotional contagion between a novice teacher and adult English learners across proficiency levels in a private language learning context, with teachers assuming recipient roles. Specifically, lower-level adult learners tended to experience anxiety and confusion due to the gap between their motivations and actual English ability. Their negative emotions were expressed through behaviors perceived by the teacher, subsequently influencing the teacher’s emotions. As students progressed to higher proficiency levels, their emotional experiences generally grew more positively due to improving language skills and relationships. The emergence of emotions like pride and joy related to achievement and belonging illustrates the intricacies of adult learning motivations rooted in practical goals like career advancement (Bigelow and Schwarz, 2010). Thus, the evolving learners’ emotions over time reciprocally shaped the novice teacher’s emotional trajectory.

Teachers in private educational institutions often occupy relatively disadvantaged positions compared to public education contexts. Private training schools prioritize student satisfaction and institutional reputation, as these factors directly impact enrollment rates and subscription renewals. When interacting with students, instructors in such training facilities unconsciously adopt additional roles beyond traditional teaching duties. That is, teachers simultaneously serve as educators delivering knowledge while attentively monitoring the emotions of their pupil audience in their capacity as service providers. This complex interplay between student emotions and teacher duties provides crucial context for elucidating the emotional contagion processes that transpire between instructors and adult English learners.

Previous research has primarily focused on the impact of teachers on students’ emotions (Moskowitz and Dewaele, 2021), with less emphasis on the influence of students on teachers. This study reveals that in adult English education, due to changes in the teacher–student relationship, students are more likely to be initiators of emotional contagion, while teachers become recipients. According to the cognition-arousal theory, emotions are the integrated result of environmental factors, cognitive processes, and physiological states in the cerebral cortex (Reisenzein, 1983). For novice learners, the incongruity between strong motivation and weak English foundations can lead to negative emotions such as anxiety and confusion. These negative emotions not only adversely affect the students’ English learning (Horwitz, 2001), but also influence the teacher’s emotions. The mainstream mechanism in emotional contagion, the imitation-feedback mechanism, posits that people tend to imitate others’ emotional expressions, including facial expressions, intonation, posture, and gestures (Hatfield et al., 2014).

The CVT of academic emotions (Pekrun, 2006) provides a critical framework for understanding these dynamics. CVT posits that emotions in educational contexts arise from the interaction between individuals’ perceived control over learning activities and the value they place on these activities. In this study, adult learners’ strong motivation to achieve practical goals, such as career advancement or studying abroad, reflects high task value. However, their weak foundational skills often diminish their perceived control, resulting in anxiety and confusion, particularly at lower proficiency levels. These negative emotions align with Pekrun’s (2006) assertion that low control combined with high value generates adverse emotional states, which can impede learning and motivation.

In this adult English training institution, most students range between 25 and 45 years old with professional experience. These adults pursue English training for diverse career goals and hold high expectations for teachers. After investing significant financial and time costs, adult English learners hope to improve their English proficiency quickly, achieve the advertised learning outcomes, and thereby “find better employment” or “study abroad by passing exams.” The control-value theory explains that when learners experience a discrepancy between their expectations and their actual progress, they are more likely to feel a lack of control, leading to anxiety or frustration. Teachers perceive these emotions through classroom interactions, further emphasizing the bidirectional nature of emotional contagion.

Over time, as students’ proficiency improves, they gain greater perceived control over their learning outcomes, fostering positive emotions like pride and joy. For instance, students expressed pride when correctly answering questions or participating in speech competitions. These positive emotional shifts align with the CVT’s proposition that increased control enhances enjoyment and achievement-related pride (Pekrun and Perry, 2014). Students also regulate their emotions by reassessing their knowledge and learning states, reducing the impact of negative emotions through cognitive reappraisal (Gross, 2002). For example, as students built stronger relationships with peers and teachers, they reported feeling a “sense of home,” which contributed to their emotional stability and motivation.

### 5.2 Emotional labor of adult English teachers: navigating challenges and managing emotional dynamics

Emotional labor refers to the process of managing emotions and expressions to meet emotional requirements in the workplace (Hochschild, 2010). For English teachers, emotional labor involves the emotional regulation behaviors and discourse practices carried out to reconcile conflicts between rules, ideologies, and practices (Benesch, 2020). The emotional labor of English teachers is influenced by various factors, such as power relations in schools or institutions, their teaching philosophies, and relationships with students (Benesch, 2017, 2018). This study also found that the teacher used two emotion regulation strategies: reappraisal and suppression (Gross, 2002). Gross concluded that reappraisal is more effective emotionally, cognitively, and socially than suppression, but in some cases, suppression may be more necessary. The importance of flexibly using multiple emotion regulation strategies to adapt to different situational needs is emphasized. Genuinely expressing, as proposed by Yin (2016) in Chinese educational settings, was not observed in this study. In the adult education environment, influenced by factors such as role expectations and the teacher’s role as a service provider, the power relationship between teachers and students has shifted. Genuinely expressing may harm students and negatively affect the relationship.

In adult English training institutions, there are usually assessment requirements imposed on teachers by the institutions. Besides fulfilling daily teaching tasks, teachers are also expected to complete other tasks mandated by the institution. Both these responsibilities affect teacher assessments and performance, consuming a significant portion of an English teacher’s time. As a teacher new to the training industry, I experienced a conflict between my teaching philosophy and student needs, leading to emotional labor. Based on the school’s educational philosophy and the courses taught, I believed in using communicative teaching methods and designing various activities to enhance students’ listening and speaking skills. However, the implementation of communicative teaching faced major challenges, including the traditional teacher-centered educational culture, relatively passive learning methods, and exam-oriented approaches. These challenges align with Hochschild’s (2010) concept of surface acting, where teachers suppress their authentic emotions to meet institutional expectations and present a professional demeanor.

Rao (1996) suggests combining traditional teaching methods, such as the audio-lingual method and grammar-translation method, with communicative teaching methods, avoiding an either-or approach. In this study, communicative teaching methods were found to be unsuitable for some novice learners who “pursued language knowledge.” This mismatch between my teaching philosophy and the learners’ preferences required deep acting—an effort to align my internal feelings with externally displayed emotions (Hochschild, 2010). For instance, while I was frustrated after investing significant effort with only partial student engagement, I sought to empathize with the learners’ perspectives and adjust my methods accordingly. Despite changing textbooks and teaching methods after the incident, I did so reluctantly, undergoing inner struggles and compromises.

Emotion regulation strategies also played a crucial role in managing emotional labor. Gross’s (2002) framework of emotion regulation highlights strategies such as cognitive reappraisal and suppression. Cognitive reappraisal involves reframing situations to reduce their emotional impact, which I employed by viewing student feedback as constructive rather than personally critical. For example, a student’s comment about preferring grammar-focused lessons over activities pushed me to rethink the balance between communicative and traditional methods. Suppression, on the other hand, was evident in interactions where I concealed frustration and maintained a supportive demeanor, as encouraged by institutional expectations (Richards, 2022).

Although teachers are encouraged to display positive emotions, such as enthusiasm and patience, while suppressing negative ones, this can create internal tension (de Ruiter et al., 2021; Richards, 2022). Emotional labor requires balancing these expectations with authentic emotional experiences. In my case, the tension between my idealized teaching philosophy and the pragmatic needs of students highlighted the complexity of emotional labor in adult education. While moments of frustration and disappointment were inevitable, they were counterbalanced by the intrinsic rewards of teaching, such as fostering student confidence and witnessing their growth.

### 5.3 Teacher–student relationships in adult education: emotional dynamics and shifting norms

The teacher–student relationship in traditional Chinese education is heavily influenced by cultural and systemic factors, resulting in a hierarchical structure where teachers hold authority and students are expected to exhibit obedience. Scholars in the field argue that this hierarchy is maintained to ensure discipline and uphold the teacher’s authority in the classroom (Rao and Chen, 2020). Although efforts to foster harmonious relationships have improved the dynamic (Liu, 2015), the power distance created by this hierarchy remains stable (Murray et al., 2020). The Chinese education system, characterized by the exam-oriented approach, large class sizes, and Confucian values, reinforces this unequal relationship (Lyu, 2021; Ma, 2021). These factors collectively shape the authority-obedience dynamic that is deeply ingrained in traditional school settings, such as secondary education.

In contrast, the context of adult education, as explored in this study, diverges significantly from these traditional norms. Adult education schools typically lack the exam pressures that dominate traditional educational settings. Smaller class sizes and the closer age proximity between teachers and students further dilute the traditional power hierarchy. In this study, the “authority-obedience” relationship was challenging to enforce, as students did not conform to expected behaviors, such as memorizing vocabulary or attending evening self-study sessions on time. As a new teacher and head teacher, I often found myself feeling helpless when students disregarded instructions that would have been readily followed in a traditional school environment.

The shift in the teacher–student relationship is also influenced by the changing roles and expectations in adult education. Unlike traditional settings, where teachers are primarily authority figures, teachers in adult education often assume roles akin to service providers. Adult learners, as fee-paying clients, possess a certain level of influence over the teacher’s career survival. Students’ opinions can directly impact on institutional evaluations of teachers, as supervisors are quick to address complaints or feedback. For instance, in this study, after three students changed classes, the school supervisor immediately sought a discussion with me, creating a sense of unease and self-doubt about my teaching practices. This dynamic underscores a significant shift: teachers in adult education settings must navigate a dual role of educator and service provider, balancing professional expectations with the need to meet student demands.

In traditional Chinese primary and secondary school classrooms, teachers are generally in a dominant position, and their language and behavior often have a significant impact on students’ emotions. Both teachers and students are emotional initiators and receivers, influencing and “infecting” each other. However, in the adult education context of this study, the dynamic shifts: teachers, aiming to maintain good relationships with students, often become the recipients of emotions. From the fact that adult students are “uncooperative” (not studying as required by the teacher) and that teachers avoid using the common “pretending to be angry” strategy seen in primary and secondary schools, it is clear that teachers in this context tend to avoid influencing students’ behavior through negative emotions. According to the control-value theory, adult students, having invested significant time and money in learning English, express their emotions more obviously and urgently as beginners. As a result, in the adult education environment, influenced by factors such as role expectations and the teacher’s position as a service provider, the power relationship between teachers and students has shifted slightly compared to traditional secondary school classrooms. Teachers are more likely to become the recipients of students’ emotional contagion, a phenomenon where students’ emotions come first, followed by teachers’ emotions. Conversely, adult students exhibit unique behaviors that differ from traditional students. Despite their confusion and discontent with a teacher’s approach, they often avoid direct confrontation or complaints to supervisors, likely influenced by Chinese cultural norms such as face-saving. Instead, students in this study tended to transfer classes quietly, using this as an indirect strategy to address their discontent.

The teacher–student relationship in adult education significantly impacts teachers’ emotions, shaping their experiences of emotional labor. As demonstrated in this study, the quality of teacher–student interactions often influenced my emotional state. For example, when students expressed negative emotions such as anxiety or confusion, I experienced corresponding feelings of self-doubt and frustration. On the other hand, moments of student success, such as their progress in English proficiency or achievements in activities like speech competitions, elicited positive emotions such as pride and fulfillment. These emotional exchanges align with the concept of true emotional expression (Hochschild, 2010), where teachers genuinely share positive emotions stemming from students’ accomplishments. However, the dual role of educator and service provider compounded the emotional labor, requiring careful management of my emotional responses to maintain professionalism and meet institutional expectations. This highlights the intricate relationship between emotional labor and the evolving teacher–student dynamic in adult education setting.

### 5.4 Implications and limitations

This study highlights the importance of teachers being prepared to address the negative emotions expressed by adult English learners. Teachers can use successful student cases to motivate others, help learners understand the challenges of foreign language learning, encourage perseverance, and discourage unrealistic expectations of rapid success. Inherent learning habits and methods of adult learners, such as using Chinese associations for word memorization, should not be indiscriminately criticized or dismissed. These methods have both advantages and disadvantages, and teachers should encourage learners to compare new and traditional approaches, empowering them to find what works best. By understanding the interplay of control and value, teachers can provide targeted support, fostering both emotional stability and academic growth in adult learners.

The findings further suggest that teachers play a pivotal role in shaping learners’ perceptions of control and value. Effective teaching strategies, such as recognizing and celebrating students’ efforts, providing relatable examples, and creating structured learning environments, can significantly enhance learners’ sense of control. For instance, integrating explicit grammar instruction alleviated learners’ confusion in this study, supporting Pekrun’s (2006) argument that clear and structured guidance bolsters perceived control and mitigates negative emotions. By cultivating positive emotional experiences, teachers can inspire persistence and resilience in adult learners, fostering a productive learning environment.

Additionally, teachers should strive to balance the traditional knowledge-centric needs of adult novice learners with communicative teaching methods. Classroom activities should be engaging yet manageable, designed to build confidence without overwhelming learners. Teachers should also develop emotional regulation strategies to effectively manage the challenges of emotional labor, such as conflicting teaching philosophies or tense student relationships. Strategies like cognitive reappraisal, empathy, and reflective practice can help teachers navigate these challenges while promoting positive emotional interactions. By integrating these approaches, educators can address the complexities of emotional labor, enhancing the overall effectiveness and harmony of the learning environment.

While this study provides valuable insights into the emotional dynamics between teachers and adult English learners, several limitations should be noted. First, the study relies on qualitative data from a single private language training institution, limiting the generalizability of the findings to other contexts or educational settings. Second, the self-narrative methodology introduces potential biases, as the teacher-researcher’s perspectives may influence data interpretation. Third, the sample size of 10 adult learners, though sufficient for qualitative inquiry, restricts the breadth of perspectives represented. Future research could address these limitations by incorporating longitudinal designs, larger sample sizes, and comparative analyses across different educational contexts to provide a more comprehensive understanding of emotional contagion in adult language education.

## 6 Conclusion

The concept of emotional contagion, as established by Hatfield et al. (1993), frames emotions as a process of automatic mimicry and synchronization that leads to emotional convergence between individuals. While this framework has encouraged extensive research on emotional dynamics in educational settings, it also highlights the need for more empirical studies exploring the complexities of cultural, institutional, and role-specific factors in shaping emotional contagion. The complexity and particularity inherent to qualitative research often make such studies difficult to undertake. However, the unique context of this study offers a rare and valuable opportunity to investigate emotional contagion in depth.

This study reexamines traditional assumptions by highlighting the pivotal role of adult learners as emotional initiators in shaping teacher–student emotional dynamics. In the context of traditional Chinese primary and secondary school classrooms, teachers typically occupy a dominant position, with their language and behavior significantly influencing students’ emotions. Both teachers and students act as emotional initiators and receivers, mutually “infecting” each other. However, in the adult education context of this study, the dynamic shifts dramatically. As a novice teacher without prior experience teaching adults, I found myself particularly susceptible to emotional contagion. Students’ emotions, such as anxiety, confusion, or discontent, often “infected” me, intensifying my self-doubt, disappointment, or anger. This heightened sensitivity was exacerbated by factors such as cultural norms that discourage overtly negative emotional displays, institutional pressures to maintain positive teacher–student relationships, and the avoidance of strategies like “pretending to be angry,” which are common in primary and secondary school teaching.

Within the context of a private language training institution in China, the findings reveal how adult learners—motivated by career advancement, personal growth, and high expectations—subtly initiate emotional exchanges through culturally embedded behaviors influenced by norms such as “face-saving.” These emotions, both positive and negative, are frequently understated but have a significant impact on teacher behavior, emotional regulation, and pedagogical strategies. For the novice teacher in this study, this creates a dual burden of emotional labor and self-regulation as they navigate institutional pressures and student expectations while simultaneously refining their teaching practices through reflective teaching logs as a method of reappraisal.

Looking forward, these findings have broader implications for the field of emotional contagion and adult education. By situating emotional contagion within the specific cultural and institutional context of private language training in China, this study not only expands the theoretical scope of the concept but also provides actionable insights for educators and researchers. Teachers can draw on strategies such as sharing success stories, promoting perseverance, and tailoring teaching methods to diverse learner needs to mitigate negative emotions and foster positive emotional exchanges. Future studies could explore how emotional contagion operates across different cultural backgrounds, professional settings, and educational systems to further refine its theoretical framework.

## Data Availability

The original contributions presented in the study are included in the article/supplementary material, further inquiries can be directed to the corresponding author.
